# **Biomechanical testing of lateral and medial ligament reconstruction using bone anchors with autologous resident tissue: influence of different reconstruction techniques on stability and mobility**

**DOI:** 10.1007/s00402-025-06004-6

**Published:** 2025-07-28

**Authors:** Lea Marie Keßling, Anna Altemeier, Dennis Nebel, Sarah Ettinger, Kiriakos Daniilidis, Christian Plaaß, Christina Stukenborg-Colsman, Leif Claaßen

**Affiliations:** 1 Department of Orthopaedic Surgery ‑ DIAKOVERE Annastift, Hannover Medical School, Hannover, Germany; 2 University Hospital for Orthopedics Pius Hospital Oldenburg, Oldenburg, Germany; 3 Laboratory for Biomechanics and Biomaterials, Department of Orthopaedic Surgery ‑ DIAKOVERE Annastift, Hannover Medical School, Hannover, Germany; 4 Orthopedics Trauma Center (OTC) Regensburg, Regensburg, Germany; 5 Orthoprofis Hannover, Hannover, Germany

**Keywords:** Ligament repair, Anterior talofibular ligament, Deltoid ligament, Medial instability, Chronic ankle instability, Biomechanics

## Abstract

**Introduction:**

The optimal surgical technique for chronic ankle instability remains disputable. This study had two main questions: the effect of additional medial collateral ligament (MCL) stabilisation in ankle instability and comparison of open and arthroscopic surgical techniques for lateral ligament repair.

**Materials and methods:**

We used 10 fresh-frozen cadaver feet (Science Care, Phoenix, AZ, USA) per group. Eight states were tested in an open surgery group: native; MCL cut; MCL repair; anterior talofibular ligament (ATFL) cut; calcaneofibular ligament (CFL) cut; MCL re-cut + ATFL repair; MCL re-cut + AFTL and CFL repair; and MCL, ATFL, and CFL repair. Three states were tested in an arthroscopic group: Native; ATFL cut; and ATFL repair. A multidirectional loading test with five different movements (anterior translation (AT), supination (SUP), pronation (PRO), internal rotation (IR), and external rotation (ER)) was performed using a robotic system with six degrees-of-freedom.

**Results:**

Refixation of the ATFL and CFL resulted in significant reductions in AT, IR, and SUP laxities (*p* < 0.05). Compared to this we observed a significant reduction of ER and PRO laxity when the MCL was additionally repaired (“MCL, ATFL and CFL repair”) (*p* < 0.05). The outcomes of “MCL, ATFL and CFL repair” of the open procedure showed no significant differences in AT, SUP and IR laxity in the different ankle positions to the outcomes of “ATFL repair” of the arthroscopic procedure (*p >* 0.05).

**Conclusion:**

Lateral ligamentoplasty leads to stabilisation of the ankle joint in AT, IR and SUP. Additional medial stabilisation resulted in further stabilisation, highlighting the relevance of preoperative and intraoperative evaluations of the medial ankle ligaments treating ankle instability. The stabilisation of the ankle joint by open and arthroscopic techniques was comparable for lateral ligament repair.

## Introduction

Ligamentous lesions of the talofibular joint are one of the most common injuries in humans [1, 2]. Approximately 20% of ankle osteoarthritis are caused by chronic ankle instability (CAI) [3–5]. Consequently, sufficient ankle stabilisation may be relevant to prevent ankle osteoarthritis [5, 6].

An open procedure with ligamentoplasty remains the treatment of choice for CAI after failed conservative therapy. Operative options include an internal suture of the ligament or a bony refixation via a bone anchor. A distinction must be made between anatomical and non-anatomical reconstruction. An anatomical reconstruction is a procedure that aims to restore the original insertion and orientation of the ligaments. This approach is believed to preserve normal joint biomechanics. It is therefore considered a standard of care. Tenodesis as a non-anatomical variant should be avoided due to worse long-term results [7]. Tightened ligament reconstruction using bone anchors has demonstrated satisfactory clinical results [8]. The reconstruction of the affected anterior talofibular ligament (ATFL) and/or calcaneofibular ligament (CFL) is a common procedure for lateral ankle instability [8].

The medial collateral ligament (MCL) consists of superficial and deep layers with up to eight different ligaments: Tibionavicular ligament, fibers to spring ligament, tibiocalcaneal ligament, superficial posterior tibiotalar ligament, anterior tibiotalar ligament, deep posterior tibiotalar ligament, deep to tibiocalcaneal ligament and posterior to sustentaculum tali ligament [9, 10]. Typically, superficial layers of the MCL are affected in CAI [11–13]. The MCL stabilises the ankle against external rotation, pronation and plantarflexion [4, 5, 14]. Retrospective studies with small sample sizes showed good clinical results after medial and lateral ligament repair [15, 16]. However, the stabilising effect of ligamentoplasty on medial and rotational instabilities remains mostly unclear. Especially the biomechanical evidence is missing.

Minimally invasive endoscopic ligamentoplasty has become increasingly popular in recent years. The advantages are reduced soft tissue trauma, smaller scars, lower rates of wound complications, and by trend earlier postoperative recovery [17, 18]. Previous studies have reported that the results of this procedure are clinically comparable to those of open procedures [19]. However, biomechanical studies on this topic are lacking.

This biomechanical study investigated the effect of medial ligamentoplasty on ankle joint stability, particularly rotational stability. Furthermore, we assessed the effects of lateral ligamentoplasty in an endoscopic technique. Finally, we compared the endoscopic and open procedures.

## Materials and methods

### Specimen preparation

The local ethics committee approved this study (approval no. 8563_BO_K_2019). Twenty frozen cadaver feet (Science Care, Phoenix, Arizona, USA) with lower legs to toe-tip were used; Eight feet were from women and twelve were from men, with an average age of 54 (range, 39–65) years. The specimens were stored at − 20 °C and thawed overnight. All tests on each specimen were performed on the same day and the specimens were periodically sprayed with 0.9% NaCl to avoid tissue damage.

The specimens were randomly assigned to two groups (open surgery and arthroscopic group). The group size was determined in orientation to previous biomechanical studies with similar designs [20–23]. Observations and radiography revealed no severe deformities, trauma, scars, or signs of previous trauma. No previous surgical procedures were performed on the upper ankle joints.

**Fig. 1:**
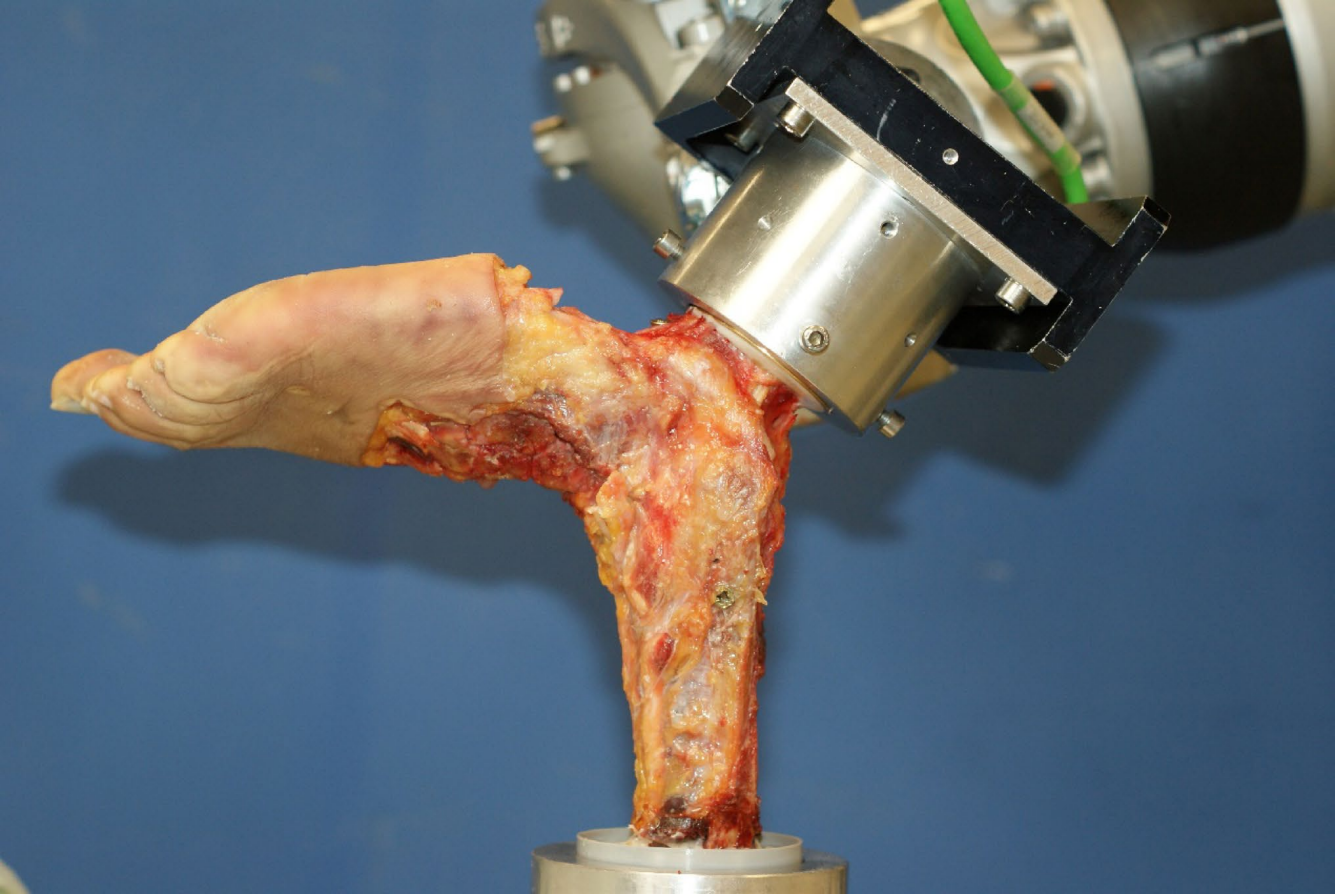
Test-setup. Specimens in the native state. The tibia was fixed to the testing rig, and the calcaneus was attached to the end effector of the KUKA KR 16 − 2 robot (KUKA AG, Augsburg, Germany)

The soft tissue around the lateral and medial malleoli was carefully removed in the open surgery group, and the ATFL, CFL, and MCL were exposed without damaging any ligamentous structures. No preexisting damage to any of the ligaments was observed during the preparation. The distal tibiofibular syndesmosis was fixed with a screw, and the subtalar joint was fixed with two crossing screws, according to previous studies [20, 24–26]. Fixing the subtalar joint with screws could theoretically restrict the normal function of the CFL. However, in our experimental setup, subtalar joint fixation was necessary to specifically assess the mechanical performance of the lateral ankle ligament repair under standardized conditions, minimizing confounding subtalar motion. Additionally, by adapting the methodology of previous studies we enhance comparability [20, 24–26]. The proximal portions of the tibia and fibula were embedded in a two component casting polyurethane resin (Rencast FC52/53; Huntsman Corp, Woodlands, TX, USA) in a metal sleeve and mounted on the testing rig. The calcaneus was embedded and attached to the force and moment sensors of the robotic system using a custom-made mounting adapter (Fig. 1).

### Surgical procedure

**Fig. 2:**
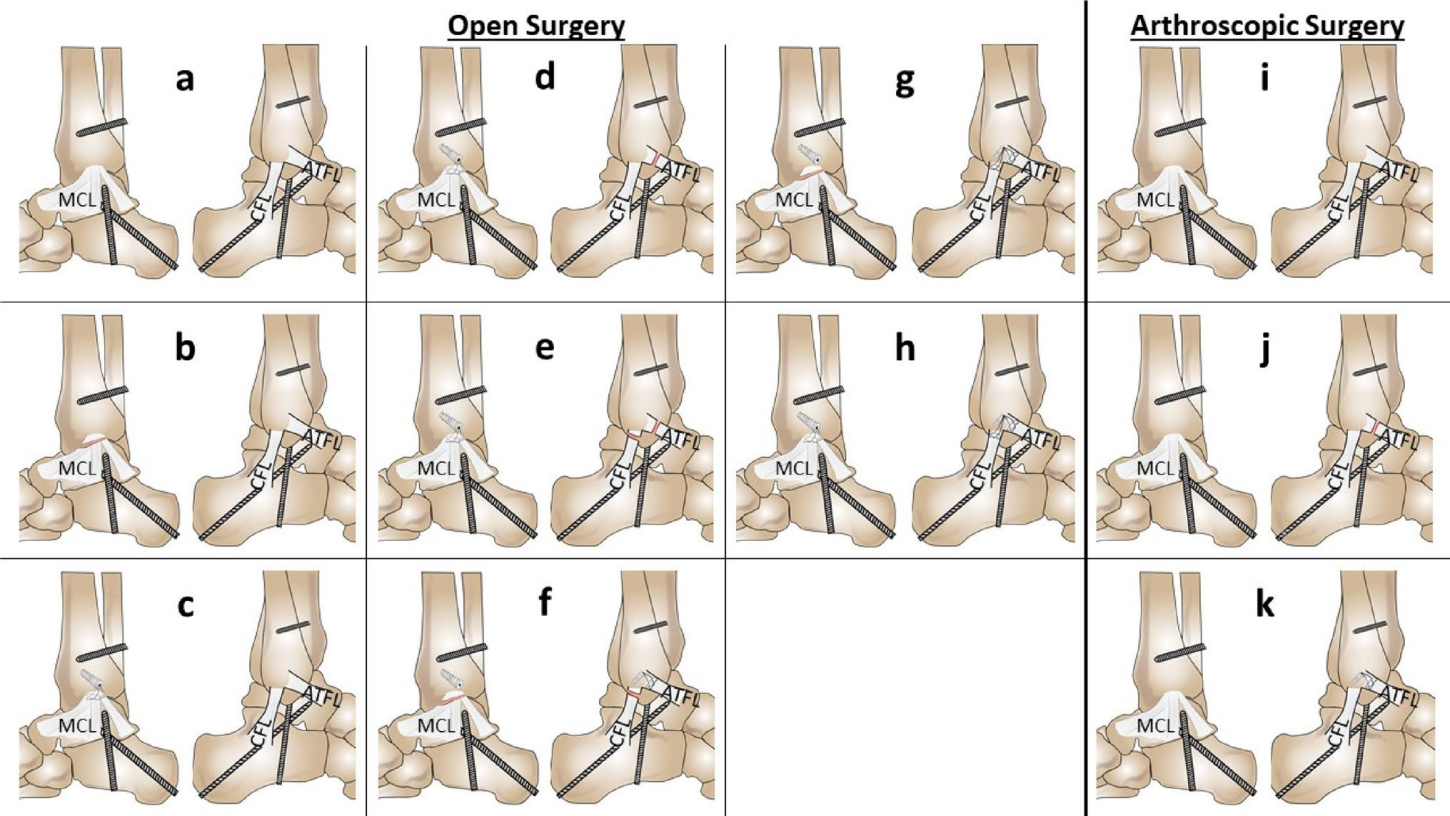
Surgical procedure. Detailed illustration of the precise sequence of the surgical procedure. The left half of the figure shows the sequence of the open procedure. MCL + ATFL + CFL native (**a**); MCL cut, ATFL + CFL native (**b**); MCL repair, ATFL + CFL native (**c**); MCL repair, ATFL cut, CFL native (**d**); MCL repair, ATFL + CFL cut (**e**); MCL recut, ATFL repair, CFL cut (**f**); MCL recut, ATFL + CFL repair (**g**); MCL re-repair, ATFL + CFL repair (**h**). The right half of the figure illustrates the sequence of the arthroscopic procedure. MCL + ATFL + CFL native (**i**); MCL + CFL native, ATFL cut (**j**); MCL + CFL native, ATFL repair (**k**). MCL, medial collateral ligament; ATFL, anterior talofibular ligament; CFL, calcaneofibular ligament

The specimens in the open surgery group were tested under eight different states (Fig. 2):

- Native State (MCL + ATFL + CFL native).
- Resection of MCL (MCL cut, ATFL + CFL native).
- Refixation of MCL (MCL repair, ATFL + CFL native).
- Resection of ATFL (MCL repair, ATFL cut, CFL native).
- Resection of CFL (MCL repair, ATFL + CFL cut).
- Resection of MCL repair, ATFL repair (MCL recut, ATFL repair, CFL cut).
- CFL repair (MCL recut, ATFL + CFL repair).
- Re-Refixation of MCL (MCL re-repair, ATFL + CFL repair).

All ligaments were resected close to the bone of origin, respectively. This imitates the injury of the ligaments in CAI [11]. For example, it has been shown that the lateral ligament injuries that lead to CAI are those that are close to the fibular attachment [27]. The cut of MCL included the superficial parts of the ligament following Panchani (Tibiospring ligament, tibionavicular ligament, tibiaocalcaneal ligament and the posterior superficial tibiotalar ligament) [9]. This represents the affected parts of MCL in CAI [12, 13]. The puncture sites for the reconstruction were performed at a defined distance of 5 mm from the ligaments origins. ATFL and CFL were repaired with different pairs of sutures of the same anchor. The Re-Refixation of the MCL was done with a second pair of sutures of the same anchor.

The specimens in the arthroscopic group were tested under three states (Fig. 2):

i) Native state (MCL + ATFL + CFL native).
j) Resection of ATFL (MCL + CFL native, ATFL cut).
k) Refixation of ATFL (MCL + CFL native, ATFL repair).

**Fig. 3:**
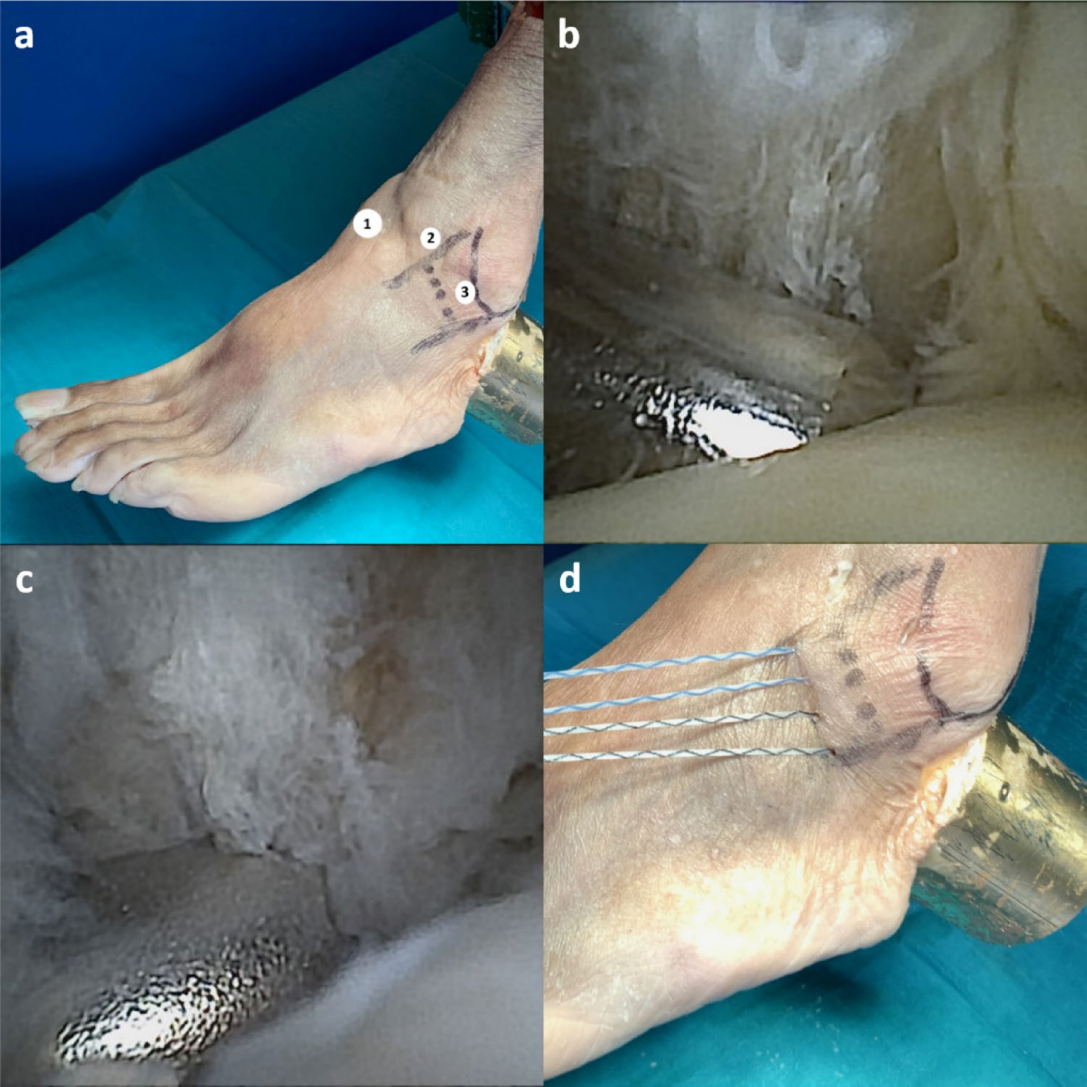
Arthroscopic portals (**a**): Three portals for surgery in the arthroscopic group. 1 medial portal, 2 anterolateral portal, and 3 lateral portal. The safe zone was drawn with the distal fibula, superior margin of the peroneal tendons, and intermediate branch of the superficial peroneal nerve. Arthroscopic view (**b**): The shaver is used to perform the resection of the ATFL, anterior talofibular ligament. Arthroscopic view (**c**): The sleeve of the anchor was placed at the origin of the ATFL, anterior talofibular ligament, where the anchor was inserted into the fibula. Refixation of the ATFL, anterior talofibular ligament. Outlet of the suture pairs within the safe zone (**d**)

Arthroscopic surgery was performed using three portals: medial, anterolateral, and lateral (Fig. 3a). A 2.0 mm arthroscopic system (NanoScope; Arthrex, Naples, FL) was introduced through the medial portal, and the instruments were inserted through the anterolateral portal [17, 19, 28]. The 2.0 mm arthroscopic system was shown to provide good visualization and safety of surgery [29]. A 3.8 mm shaver (Arthrex, Naples, FL) was used to create the state “MCL + CFL native, ATFL cut” (Fig. 3b).

The anchor was introduced through the anterolateral portal (Fig. 3c), and ATFL refixation was performed using the modified Broström technique in orientation to Acevedo et al. (Fig. 3d) [18, 30, 31].

All reconstructions were performed using a bone anchor (Selfpunching 2.6 Fibre Tak^®^ Suture Anchor, Double Loaded with 1.3 mm Suture Tape, Arthrex, Naples, FL) [32]. Ligaments were fixed to the anchor with a modified Mason–Allen stitch [33].

### Test setup

**Fig. 4:**
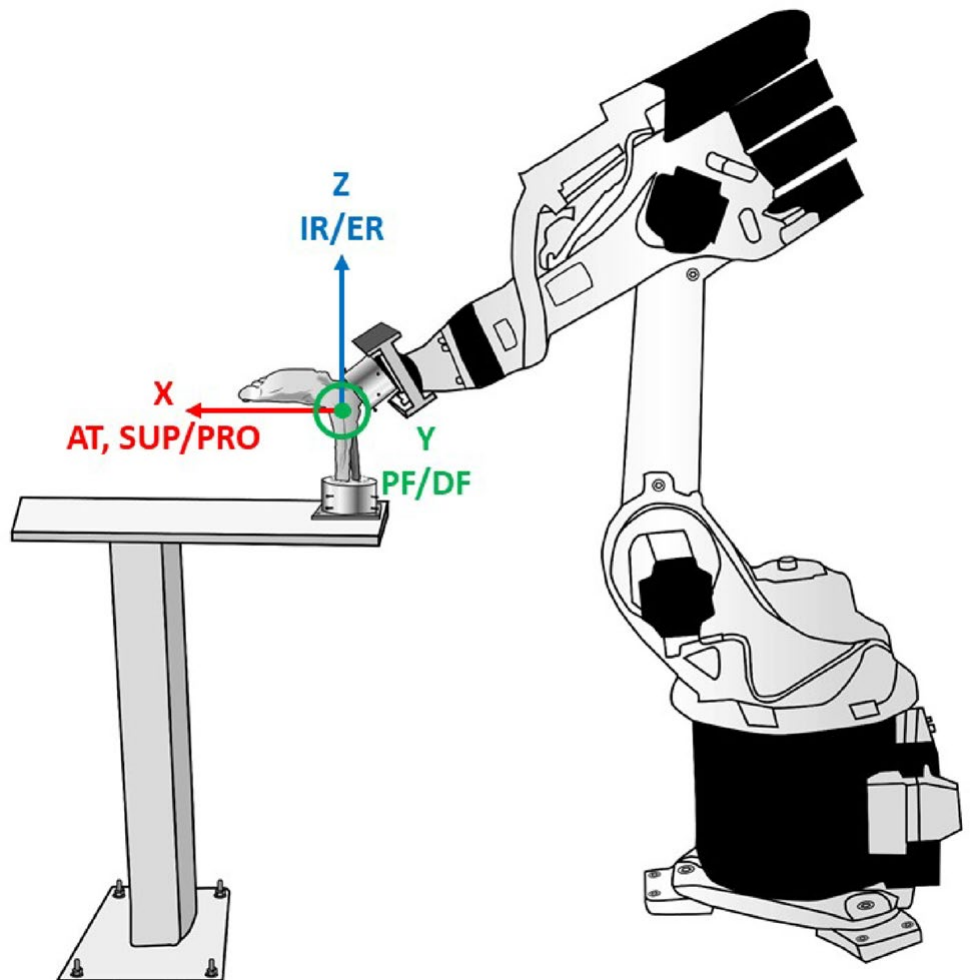
Schematic illustration of the test-setup. The illustration shows the global coordinate system and related movement directions. The x-axis with AT, SUP and PRO, the y-axis with PF and DF, and the z-axis with IR/ER are indicated. AT, anterior translation; SUP, supination; PRO, pronation; PF, plantar flexion; DF, dorsiflexion; IR, internal rotation; ER, external rotation

This setup was adapted from Shoji et al. [20]. The tibia was fixed to the testing rig, and the relative movement of the talocrural joint was performed by moving the calcaneus attached to the end effector of the robot. Testing was performed using a KUKA KR 16 − 2 robot (KUKA AG, Augsburg, Germany) equipped with a force-torque sensor (Delta Net, ATI Industrial Automation, Apex, NC, USA). The setup allowed motion control with a repeatability of 0.05 mm and measurement of forces and moments with resolutions of 0.25 N and 7.5.10^-3^ Nm, respectively (Fig. 4). The robotic system was used as a manipulator and applied unconstrained rotational moments of up to 1.7 Nm for testing the external rotation (ER), internal rotation (IR), supination (SUP), and pronation (PRO). Anterior translation (AT) was tested up to a maximum of 60 N. The total range of motion (ROM) was recorded using the robotic testing system. Laxity was defined as the movement of the calcaneus with respect to the talus at 60 N for AT and 1.7 Nm for SUP, PRO, IR, and ER moments [20].

The global coordinate system used to detect the movements was determined as follows. The z-axis was defined as the axis aligned with the tibia and directed distally. The x-axis was directed at 90 degrees perpendicular to the z-axis along the course of the forefoot and anteriorly. Finally, the y-axis was defined as the axis orthogonal to the x- and z-axes and was directed laterally for the right sided specimens and medially for the left sided specimens. Next, a specimen-specific coordinate system at the geometric centre of the talocrural joint was defined to describe the motion of the talus with respect to the tibia and fibula as follows. The centre of rotation was determined by passive movements of the specimens by the KUKA robot. This resulted in preconditioning. Passive plantarflexion, internal-external rotation, pronation and supination were carried out using the force-moment-controlled robot prior to testing. The geometric centre was defined as the point at which the talus moved the least [32, 34–36]. The specimen-specific coordinate system was oriented codirectionally to the global coordinate system after centring the talocrural joint with the foot in the neutral position (Fig. 4).

The AT, SUP, PRO, IR, and ER were tested in each state and group. Each test was performed in four ankle positions: neutral position (NP), 15 degrees dorsiflexion (DF), and 15 degrees and 30 degrees plantarflexion (PF).

### Statistical analysis

Data collection and analysis were performed using R (version 4.3.2) in R studio (2023.09.1 Build 494). A linear mixed-effects model with the specimen as the random effect, and the group (arthroscopic and open) and the condition as the fixed effects was used to compare differences in laxity. A post-hoc analysis was performed using pairwise comparisons of the estimated marginal means with Bonferroni correction. The significance level was set at α = 0.05. The differences within the arthroscopic and open group and the interaction effects between these two groups were tested.

## Results

**Fig. 5:**
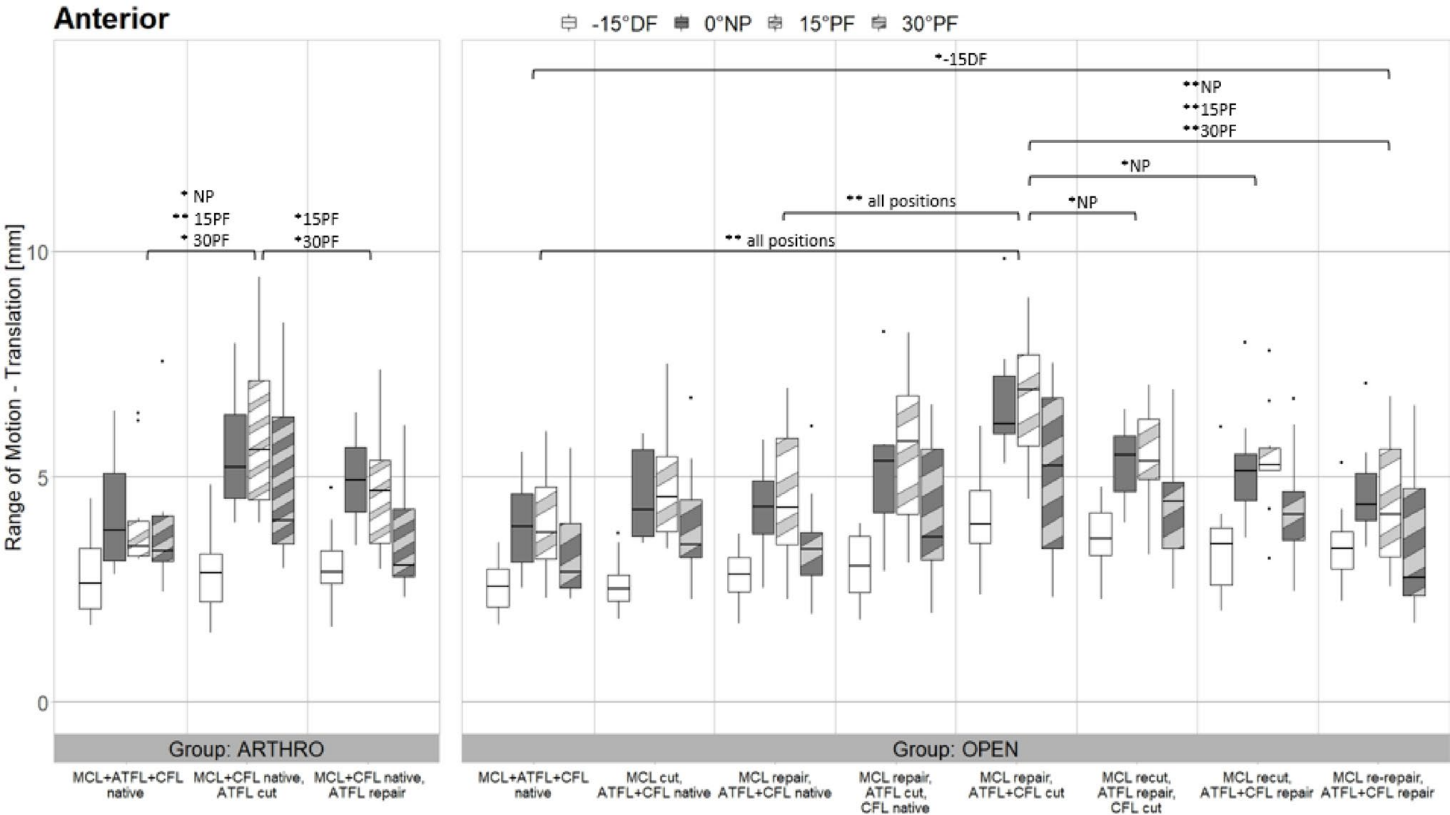
Results of Anterior Movement. Boxplots showing the Range of Motion in mm for all tested conditions in the arthroscopic group (left side) and the open group (right side) in all four ankle angle positions under Anterior loading. Brackets show significant differences between the relevant states. The symbol * indicates significant differences with *p* < 0.05 whereas the symbol ** indicates significant differences with *p* < 0.001. The significant differences are specified for the various positions (-15DF, NP, 15PF, 30PF). MCL, medial collateral ligament; ATFL, anterior talofibular ligament; CFL, calcaneofibular ligament; DF, dorsiflexion; NP neutral position; PF plantarflexion

**Fig. 6:**
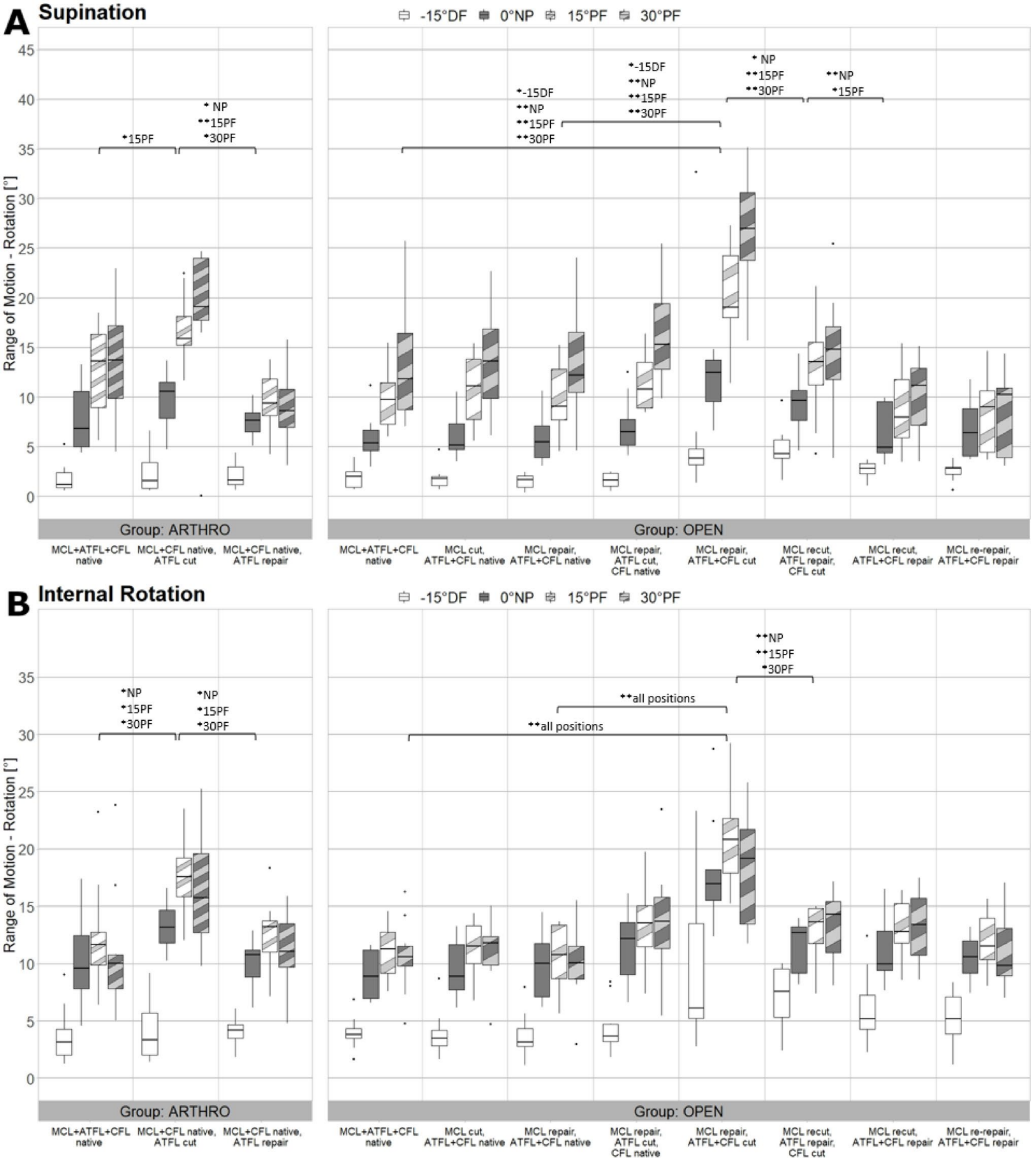
Results of Supination and Internal Rotation Movement. Boxplots showing the Range of Motion in degree for all tested conditions in the arthroscopic group (left side) and the open group (right side) in all four ankle angle positions under Supination (**A**) and Internal Rotation (**B**) loading. Brackets show significant differences between the relevant states. The symbol * indicates significant differences with *p* < 0.05 whereas the symbol ** indicates significant differences with *p* < 0.001. The significant differences are specified for the various positions (-15DF, NP, 15PF, 30PF). MCL, medial collateral ligament; ATFL, anterior talofibular ligament; CFL, calcaneofibular ligament; DF, dorsiflexion; NP neutral position; PF plantarflexion

**Fig. 7:**
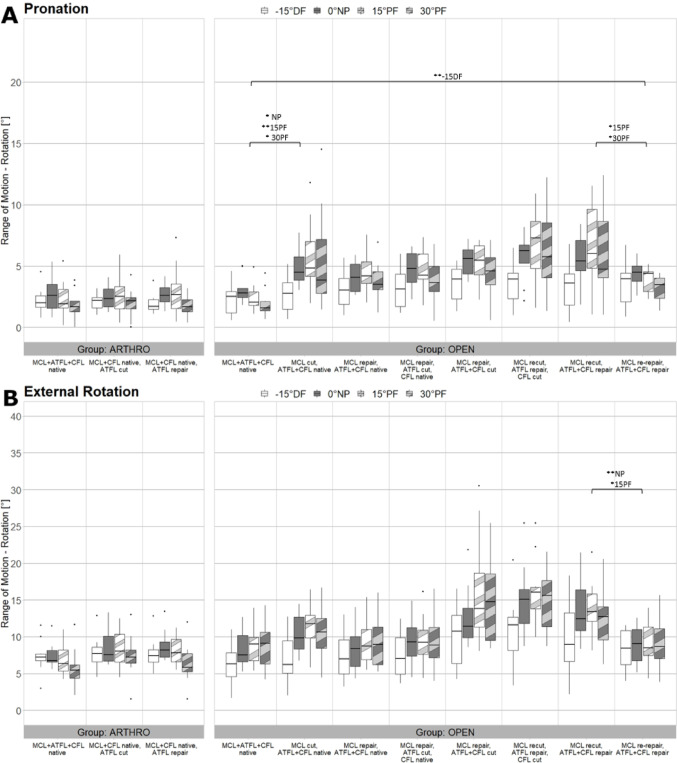
Results of Pronation and External Rotation Movement. Boxplots showing the Range of Motion in degree for all tested conditions in the arthroscopic group (left side) and the open group (right side) in all four ankle angle positions under Pronation (**A**) and External Rotation (**B**) loading. Brackets show significant differences between the relevant states. The symbol * indicates significant differences with *p* < 0.05 whereas the symbol ** indicates significant differences with *p* < 0.001. The significant differences are specified for the various positions (-15DF, NP, 15PF, 30PF). MCL, medial collateral ligament; ATFL, anterior talofibular ligament; CFL, calcaneofibular ligament; DF, dorsiflexion; NP neutral position; PF plantarflexion

Figures 5, 6 and 7 present the ROM of AT, SUP and IR, PRO and ER in 15 degrees DF, NP, 15 degrees PF and 30 degrees PF in all eight tested states. The outcomes of the open and arthroscopic groups are presented in a side-by-side manner.

### Open surgery– anterior

Resection of the ATFL and CFL resulted in significant increases of ROM in all ankle positions compared to “MCL + ATFL + CFL native” *(p < 0.001)* and also compared to “MCL repair, ATFL + CFL native” (*p* < 0.001). Refixation of the ATFL had a significant effect on the stabilisation in NP (*p* = 0.02) compared to “MCL repair, ATFL + CFL cut”. When additionally, the CFL was repaired this also showed a significant stabilisation in NP (*p* = 0.005). Also compared to this state, the followed re-repair of the MCL led to an extended significant stabilisation in NP, 15 degrees PF and 30 degrees PF (*p* < 0.001). Refixation of ATFL, CFL and MCL showed no significant differences compared to native state except for 15 degrees DF (*p* = 0.002) (Fig. 5).

### Open surgery– supination and internal rotation

Cutting ATFL and CFL showed a significant increase in SUP laxity compared to native state in all tested ankle positions (*p* = 0.04, *p* < 0.001, *p* < 0.001, *p* < 0.001) and also compared to “MCL repair, ATFL + CFL native” (*p* = 0.02, *p* < 0.001, *p* < 0.001, *p* < 0.001). Refixation of ATFL led to a significant stabilisation in NP, 15- and 30-degrees PF (*p* = 0.01, *p* < 0.001,* < 0.001).* When the CFL was additionally repaired stability increased significantly in NP and 15 degrees PF (*p* < 0.001, *p* = 0.03) (Fig. 6a).

In IR movement resection of the ATFL and CFL showed a significant increase of ROM compared to native state and also compared to “MCL repair, ATFL + CFL native” in all tested ankle positions (*p* < 0.001). Followed refixation of ATFL showed a significant stabilisation in NP, 15- and 30-degrees PF (*p* < 0.001,*p* < 0.001, *p* = 0.003) (Fig. 6b).

Refixation of ATFL, CFL and MCL showed no significant differences compared to native state in SUP and IR movement.

### Open surgery– pronation and external rotation

In PRO movement resection of the MCL led to a significant increase of ROM in NP, 15- and 30-degrees PF (*p* = 0.001, *p* < 0.001, *p* = 0.003). “MCL re-repair, ATFL + CFL repair” showed a significant stabilisation in 15- and 30-degrees PF compared to “MCL recut, ATFL + CFL repair” (*p* = 0.01, *p* = 0.04). Refixation of ATFL, CFL and MCL showed no significant differences compared to native state except for 15 degrees DF (*p* < 0.001) (Fig. 7a).

In ER movement MCL re-repair demonstrated a significant reduction of ROM in NP and 15 degrees PF (*p* < 0.001, *p* = 0.04) compared to “MCL recut, ATFL + CFL repair”. No significant difference was observed between the native state and combined MCL, ATFL and CFL repair (Fig. 7b). 

### Arthroscopic surgery

ATFL resection led to significant increase in AT laxity at NP, 15- and 30-degrees PF (*p* = 0.002, *p* < 0.001, *p* = 0.02). Refixation resulted in significant stabilisation at 15- and 30-degrees PF (*p* = 0.01, *p* = 0.02).

(Fig. 5).

When ATFL was cut, a significant increase of SUP laxity at 15 degrees PF (*p* = 0.03) was measured compared to native state. Refixation of the ATFL resulted in a significant stabilisation at NP, 15- and 30-degrees PF (*p* = 0.02, *p* < 0.001, *p* = 0.003) (Fig. 6a).

An increase of IR laxity was observed in NP, 15- and 30 degrees PF (*p* = 0.01, *p* = 0.001, *p* = 0.004) after cutting the ATFL. Refixation of the ATFL led to a significant stabilisation in NP, 15- and 30 degrees PF (*p* = 0.02, *p* = 0.003, *p* = 0.005) (Fig. 6b).

Between open and arthroscopic surgery, the analysis revealed no statistically significant differences in all tested movements and ankle positions (*p* > 0.05).

## Discussion

This study investigated the influence of medial and lateral ligamentoplasties on the stability of the upper ankle joint. These results highlight the relevance of medial ligament stabilisation in rotational instability. Furthermore, endoscopic lateral ligament repair led to results comparable to those obtained using the open technique. The experimental setup was selected based on a previous biomechanical study that compared anatomical and non-anatomical ATFL ligamentoplasty [20]. This improved the comparability and reliability of our results. The anchor fixation technique performed in our study is an established method in ligament surgery with satisfactory results in clinical studies and has already been studied biomechanically [25, 26, 30, 37–39]. The experiments were conducted using a robotic system, leading to significantly more reliable results than measurements performed by humans. Cadavers were used as test specimens. The influence of soft tissue can be considered using this system, and a closer relationship to in vivo conditions can be established compared to using bone models (e.g. sawbones^®^, Sawbone Europe, Malmö, Sweden).

By cutting the superficial parts of the MCL, the valgus tilt of the ankle and external rotation of the talus were described by cadaveric studies before [40–42]. Despite this knowledge, the need of additional medial stabilisation is currently widely debated. Retrospective studies with small sample sizes have already indicated the success of additional medial stabilisation regarding to AOFAS (American Orthopaedic Foot and Ankle Society) Score [15, 16]. Investigations of deltoid reconstruction in a biomechanical setup are lacking. As the first biomechanical study, the effect of medial ligamentoplasty on ankle stability was examined. In a simulated concomitant medial instability, a significant reduction was observed in ER at NP and 15 degree PF and PRO laxity at 15- and 30-hhh degree PF position when the MCL was additionally repaired compared to only refixation of the ATFL and CFL. The instability of the upper ankle joint significantly increased with increasing PF, whereas smaller values were observed in DF. According to this, the greatest instability in the ankle joint was measured in 30 degrees PF, whereas the values in 15 degrees DF corresponded to the highest stability. This is due to the posterior narrowing of the talus roll and relative posterior movement of the tibia on the talus during PF. Increasing PF therefore leads to an increase in mobility [14, 43, 44]. This may explain why refixation and stabilisation of the ligaments had greater effects on further movement of the foot in PF. These results highlight the relevance of medial ligament stabilisation in rotational instability.

Previous biomechanical studies evaluated the ligamentous apparatus of the upper ankle joint [20, 26]. Caputo et al. investigated the in vivo kinematics of ATFL-intact and ATFL-deficient conditions and reported an increase of 0.9 mm in AT and 5.7 degree in IR [45]. Larkins et al. reported the biomechanical effects of ATFL and CFL lesions, ATFL repair, ATFL and CFL repair, and ATFL augmentation on ankle stability [21]. From intact to an injured ATFL, they recorded an increase of 1.5 mm in AT. After they also cut the CFL, they recorded an increase of 2 mm in AT compared to the intact state. Li et al. compared the SUP angle in the intact and injured ATFL, an additional injured CFL, and the PTFL [22]. They reported an increase of 2 degree and 7 degrees when the ATFL and CFL were injured, respectively.

In the present study, AT increased by 1.5 mm, IR laxity by 3 degree, and SUP laxity by 3 degrees when the ATFL was cut. Additionally, cutting the CFL increased AT movement by 3 mm, IR laxity by 8 degrees, and SUP laxity by 7 degrees. Therefore, the results of this study are consistent with those of previous in vitro and in vivo studies. The present study underlines the biomechanical role of the lateral ligaments and effectiveness of ligamentoplasty due to an increase of laxity after cutting ATFL and CFL, respectively reduction after repair.

Furthermore, endoscopic lateral ligament repair led to results comparable to those obtained using the open technique. Clinically comparable results were obtained after comparing the open and endoscopic techniques for lateral ligament repair [28, 46, 47]. Giza et al. and Lee et al. conducted similar cadaveric studies on lateral ankle ligament repair, finding no significant differences in torque to failure, construct stiffness, or degrees to failure between open and arthroscopic techniques repair [48, 49]. However, key methodological differences exist. Both studies tested only at fixed ankle positions (15° internal rotation and 20° plantarflexion), while our study used a KUKA robotic system to evaluate multiple joint positions and moreover tested rotational instability for a more comprehensive biomechanical analysis. Additionally, neither prior study stabilized the subtalar joint or syndesmosis, potentially introducing confounding motion. Lee et al. also used different fixation methods– bone anchors arthroscopically and sutures openly– and did not fully dissect ligaments arthroscopically, possibly preserving native tension and influencing outcomes. Moreover, it should be noted that our study placed significant emphasis on the assessment of the medial ligamentous apparatus, which was not examined in any of the mentioned studies. In summary, our study offers methodological advancements over prior cadaveric work.

Cutting ATFL led to laxity in AT, SUP and IR. After arthroscopic repair no difference was shown compared to the native state in these movements. However, this study revealed no significant differences in AT, SUP, or IR laxity between open and arthroscopic ATFL stabilisation. Endoscopic repair may be a viable option for treating ankle instability, considering the reduced soft tissue damage and scar tissue development with this technique.

Our study had some limitations. First, this was a cadaveric study, which limits the transferability to living persons. Furthermore, the mean age of the cadaveric specimens was above that of a large proportion of patients with ankle ligament injuries [3]. Age and gender have an influence on the strength of ligaments. Ligamentous laxity is associated with increased age, especially in female gender. That could have affected the accuracy of the results [50]. Nevertheless, significant improvements were observed after refixation; therefore, transferability seems possible. Ligament ruptures were induced by manual cutting, which may not represent the actual condition of the ruptured ligament in vivo. However, the results of an in vivo study by Yamaguchi et al. showed only minor differences from the results of this study in terms of the measured kinematics [51]. Third, the tibiofibular and subtalar joints were fixed with screws, which may have potentially affected the results. However, this was accorded to a previous study, which increased comparability and enhanced standardisation as slight interindividual differences in additional movements of the tibiofibular and subtalar joints may have affected the results [44]. This approach has been implemented in previous biomechanical studies [20, 24–26]. Forth, the order of the set states can be questioned. Several other sequences involving cutting and repairing the lateral ligaments first were possible. Nevertheless, this is the first study to implement this protocol. This study provides relevant information, particularly regarding isolated medial instability and refixation. This highlights the need for distinct medial stabilisation in cases of additional medial instability. It may also be possible to use the endoscopic lateral ligament repair technique described by Vega et al. [28]. We did not use this technique as we wanted to use the same anchor in the open and endoscopic techniques. The technique used in this study was also implemented clinically and evaluated in a previous study [30]. Furthermore, an eight-step testing protocol implies that each specimen undergoes numerous tests in which increasing stress is applied to the tissue [22]. Nevertheless, the extended testing protocol performed using robotic testing provided new reliable information. Future studies examining endoscopic ligament reconstruction techniques as described by Vega et al. [28] or Takao et al. [52] as well as an itemized evaluation of different parts of the medial ligament complex are reasonable. It should be noted that the removal of the skin and subcutaneous tissue was performed exclusively in the open group, a procedure that was undertaken to facilitate the identification and transection of the ligamentous structure. In the arthroscopic group, the ligament was transected via the respective endoscopic portal. This discrepancy may have implications for the biomechanical stability of the ankle. Moreover, it is imperative to note that the capacity to achieve a partial cut is inherently constrained by the nature of arthroscopic surgery. However, the influence of surrounding soft tissue on the stability of the upper ankle joint is not considered to be significant. To date, no scientific studies have been found that support such an effect. Consequently, the impact of this discrepancy on the outcomes is considered to be negligible. Furthermore, the MCL was also cut and repaired in the open procedure group. Nevertheless, the stability after open and endoscopic ligamentoplasty was comparable. Biomechanical comparisons of open and endoscopic techniques are lacking. However, the clinical relevance of endoscopic ligamentoplasty is increasing. Therefore, there is a relevance of the biomechanical comparison of stability despite inhomogeneous resections in the open and endoscopic group. Additionally, a clinical study assessing stability, range of motion and functional scores as well as pain and patient’s satisfaction after endoscopic ligament repair in comparison to open ligament repair could verify the clinical transferability of the present biomechanical data.

## Conclusions

Lateral ligamentoplasty leads to stabilisation of the ankle joint in anterior translation, internal rotation and supination. Additional medial stabilisation resulted in further stabilisation, highlighting the relevance of preoperative and intraoperative evaluations of the medial ankle ligaments treating ankle instability. The stabilisation of the ankle joint by open and arthroscopic techniques was comparable for lateral ligament repair.

## Data Availability

No datasets were generated or analysed during the current study.

## Abbreviations

ATFL: Anterior talofibular ligament
CFL: Calcaneofibular ligament
MCL: Medial collateral ligament
CAI: Chronic ankle instability
AT: Anterior translation
SUP: Supination
PRO: Pronation
IR: Internal rotation
ER: External rotation
NP: Neutral position
PF: Plantarflexion
DF: Dorsiflexion
